# Defensins: A Double-Edged Sword in Host Immunity

**DOI:** 10.3389/fimmu.2020.00764

**Published:** 2020-05-07

**Authors:** Dan Xu, Wuyuan Lu

**Affiliations:** ^1^Institute of Mitochondrial Biology and Medicine, Key Laboratory of Biomedical Information Engineering of the Ministry of Education, School of Life Sciences and Technology, Xi’an Jiaotong University, Xi’an, China; ^2^Institute of Human Virology and Department of Biochemistry and Molecular Biology, University of Maryland School of Medicine, Baltimore, MD, United States

**Keywords:** antimicrobial peptide, host defense peptide, defensin, innate immunity, *Shigella*, host–pathogen interaction

## Abstract

Defensins are a major family of host defense peptides expressed predominantly in neutrophils and epithelial cells. Their broad antimicrobial activities and multifaceted immunomodulatory functions have been extensively studied, cementing their role in innate immunity as a core host-protective component against bacterial, viral and fungal infections. More recent studies, however, paint defensins in a bad light such that they are “alleged” to promote viral and bacterial infections in certain biological settings. This mini review summarizes the latest findings on the potential pathogenic properties of defensins against the backdrop of their protective roles in antiviral and antibacterial immunity. Further, a succinct description of both tumor-proliferative and -suppressive activities of defensins is also given to highlight their functional and mechanistic complexity in antitumor immunity. We posit that given an enabling environment defensins, widely heralded as the “Swiss army knife,” can function as a “double−edged sword” in host immunity.

## Introduction to Human Defensins

Defensins are a family of small (2–5 kDa), cationic host defense peptides with a β-sheet core structure stabilized by three conserved intramolecular disulfide bonds. The first mammalian defensin, also termed microbicidal cationic protein, was isolated in 1980 by Lehrer and colleagues from rabbit lung macrophages (1, 2). It was not until 1985 when the same lab discovered homologous peptides in human neutrophils did Lehrer coin the term defensin (3, 4) to describe disulfide-stabilized cationic peptides of mammalian origins with broad antimicrobial activity against bacteria, viruses and fungi. Based on disulfide topology, mammalian defensins are classified into three subfamilies, α, β, and θ-defensins (5–8). In humans, there exist only α and β-defensins. θ-defensins, with a unique circular structure stabilized by three parallel disulfide bonds in a ladder pattern, are only found in leukocytes of rhesus macaques (9). Although RNA transcripts homologous to the rhesus θ-defensin gene are found in humans, they contain a premature stop codon in the upstream signal sequence that abolishes their subsequent translation (10).

To date, six human α-defensins have been identified, which are further divided into two major classes according to their expression patterns and gene structures: myeloid defensins or human neutrophil peptides (HNPs) 1 to 4 and human (enteric) defensins (HDs) 5 and 6 (11–13). HNPs are stored in the azurophilic granules of human neutrophils, of which HNPs 1–3 and their much less abundant fourth cousin HNP4 account, collectively, for 5–7% of the total neutrophil protein (4; 14; 15). HNPs-containing granules normally undergo restricted secretion and are commonly directed for fusion with phagolysosomes, where high concentrations of HNPs directly kill phagocytosed microbes (16, 17). Upon holocrine secretion and neutrophil infiltration during inflammation, HNPs are released into the extracellular milieu through degranulation of activated neutrophils (17–19). HD5 and HD6 are constitutively expressed in and secreted by Paneth cells at the bottom of the small intestinal crypt (12, 13, 20, 21). While the concentration of HD5 at the luminal surface of the small intestine is estimated to be as high as 50–250 μg/ml, it is significantly lower at the colonic mucosal surface due to the distance from secretion (21). HD5 ranging from 1 to 50 μg/ml is also found in vaginal fluid from healthy women (22) and induced in the male and female reproductive tract in response to sexually transmitted infections (STIs) (23–25). Although more than 30 β-defensin genes exist in the human genome, only a few have been extensively characterized at the genomic and functional levels (26). Unlike α-defensin expression, which is commonly regulated at the level of secretion, β-defensin expression is transcriptionally regulated and restricted to keratinocytes of the skin and epithelial cells. For instance, while human β-defensin 1 (HBD1) is constitutively expressed, HBD2 and HBD3 are induced by microbial insults and pro-inflammatory cytokines in various epithelial and mucosal tissues (27, 28).

Since their first discovery in the early 1980s, defensins have been intensively investigated for their broad antimicrobial activities and multifaceted immunomodulatory functions under both physiological and pathogenic conditions. Many excellent reviews have shed light on a multitude of sophisticated molecular and cellular mechanisms by which defensins act against bacteria, viruses and fungi and function as pleiotropic immune effectors in inflammation, development and cancer (5, 11, 26, 29–34). By and large, defensins are heralded as the “Swiss army knife” in innate immunity against microbial pathogens. Nevertheless, accumulating recent evidence has unveiled a potential pathogenic role defensins play in host-pathogen interactions and tumorigenesis, indicating that the mechanisms of action of defensins are far more complex than previously thought. The growing recognition that defensins can be both advantageous and detrimental, depending on their spatial-temporal settings, gives us the impetus to review the recent literature on their protective and pathogenic roles in health and disease.

## Defensins in Viral Infection

**FIGURE 1:**
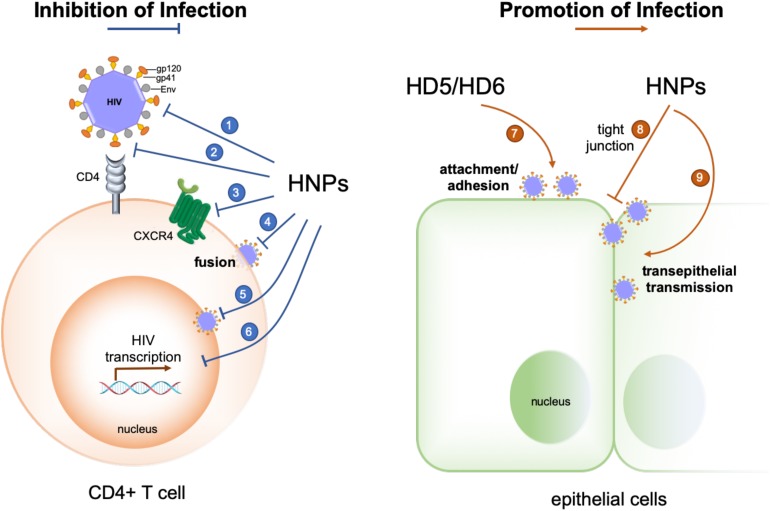
Opposing effects of human α-defensins on HIV-1 infection. Infection-inhibiting mechanisms include: (1) direct inactivation of the virus (39, 40), (2) blockade of gp120-CD4 interactions (164, 165), (3) coreceptor downregulation (41, 166), (4) inhibition of gp41- and Env-mediated viral fusion (41), (5) inhibition of nuclear import of viral RNA (30, 39), and (6) suppression of HIV transcription (30, 39, 167). Infection-promoting mechanisms include: (7) enhancing viral adhesion/attachment (25, 56, 57, 59), and (8, 9) disrupting tight junction to promote trans-epithelial transmission of HIV (60).

Defensins directly inactivate and inhibit the replication of a variety of viruses, and their multifaceted mechanisms of action have been elucidated (30, 31); the underlying mechanisms of the role of defensins in host-virus interactions are more complex as evidenced with HIV-1 (Figure 1). Early studies demonstrated that defensins are able to target multiple steps of host-virus interactions to reduce the infectivity of both enveloped and non-enveloped viruses. HNP1–3, HD5 and retrocyclins 1 and 3 deduced from human θ-defensin pseudogenes effectively block adhesion of enveloped herpes simplex virus 2 (HSV-2) to host cells by preventing HSV-2 gB interactions with its receptor HSPGs (35–37). Defensins also inhibit fusion of virions of several enveloped viruses with their host cells. Retrocyclin 2 and HBD3 interfere with viral fusion mediated by influenza virus hemagglutinin (HA) and other viral proteins such as baculovirus gp64 and Sindbis virus E1 protein (38). While HNP1 is well recognized for its direct anti-HIV activity (39, 40), it also restrains HIV-1 uptake by inhibiting Env-mediated viral fusion and downregulating host cell surface expression of CD4 and coreceptor CXCR4 (41), a controversial mechanism for HBD2 and HBD3 inhibition of HIV-1 infection (42–44).

Post-entry inhibition of viral infection by defensins has been observed with several families of non-enveloped viruses, notably HPV (45). Without affecting the binding and entry steps, α-defensins effectively block intracellular uncoating of HPV and its escape from cytoplasmic vesicles by stabilizing its viral capsid structure to prevent interactions of viral proteins and genome with host factors essential for productive infection (45–49). This general inhibitory mechanism has been verified for other non-enveloped viruses such as human adenovirus (HAdV) and JC polyomavirus where α-defensins stabilize viral capsid proteins, thus diminishing subsequent intracellular infection (50–54). Of note, post-entry inhibition of enveloped viruses such as HIV-1 and influenza by HNP1 is mediated through interfering with cell signaling pathways such as PKC that are required for viral replication (39, 55).

More recent studies, mostly by the same research groups who demonstrated the beneficial role of defensins in controlling viral infection, unveil infection-promoting effects of defensins in HIV-1 and certain serotypes of HAdV infections (25, 51, 56–60). Chang and colleagues reported that HD5 and HD6, induced by *Neisseria gonorrhoeae* infection in a cervicovaginal tissue culture system, increase HIV infectivity in a CD4- and HIV coreceptor-independent manner (25). HD5 and HD6 promote HIV infection by acting on the virion to enhance viral attachment to its target cells (57). These defensins antagonize anti-HIV activity of polyanion microbicide candidates that block HIV entry (56). HNP1, the prototypic α-defensin extensively studied for its multifaceted anti-HIV activity, is also capable of disrupting epithelial integrity to promote HIV traversal across epithelial barriers, thus facilitating viral infection and dissemination (60). These findings by the Chang group are of particular interest since increased HNP1 and HD5 expression in the genitourinary tract upon STIs could potentially generate sufficiently high concentrations of defensins to enhance HIV-1 infection under physiological conditions. Other examples regarding the enhancing effect of defensins on enveloped virus infection have been reported. For example, cryptdin 3, one of several mouse α-defensins expressed in the small intestine (61) also enhances HIV infection *in vitro* presumably by facilitating viral entry (58). A recent study shows that an alphaherpesvirus, equine herpesvirus type 1, is resistant to equine β-defensins 2–3, which inhibit bacteria and viruses, and exploits these defensins to invade the host for viral spread (62).

HNP1- and HD5-promoted viral infection has also been observed with certain serotypes of HAdV as reported by the Smith group (51), who previously deciphered the capsid-stabilizing mechanism of defensins against HPV and HAdV and delineated their structural determinants of antiviral activity (46–54, 63). As is the case with HIV-1, HNP1- and HD5-dependent enhancement in infection by HAdV-D and -F correlates with increased viral attachment to target cells independently of receptor binding (51). To address the physiological relevance of defensin-enhanced adenovirus infection, Smith and colleagues utilized a murine enteric organoid (enteroid) to examine the impact of naturally secreted cryptdins on the infectivity of an enteric mouse pathogen, mouse adenovirus 2 (MAdV-2). MAdV-2 infection increases in the enteroids expressing mouse α-defensins but not in the ones devoid of them (64). This *ex vivo* study demonstrates that α-defensin-enhanced viral infection occurs not only in traditional cell cultures, but also under physiologic conditions.

## Defensins in Bacterial Infection

Defensins are capable of killing bacteria or inhibiting bacterial growth through a multiplicity of antimicrobial mechanisms such as direct membrane disruption (11, 65, 66) and inhibition of bacterial cell wall synthesis (67–69). Defensins can also reduce bacterial infection by neutralizing secreted toxins (70–73). In general, human α-defensins are less cationic but more hydrophobic than β-defensins, and they can differ mechanistically in the killing of bacteria (11). While HBD1 and HBD2 are active preferably against Gram-negative bacteria (74), their significantly more cationic counterpart HBD3 is potently bactericidal against both Gram-positive and -negative strains (75). Due to its heavily cationic nature, HBD3 broadly kills bacteria in a structure-independent manner (76, 77). Notably, disulfide reduction of the weakly bactericidal HBD1 turns it into a potent antimicrobial peptide against opportunistic pathogenic fungi and Gram-positive commensal bacteria (78). Excellent reviews on the antifungal activity of defensins are also available (79, 80). Our review focuses on the role of human α-defensins in host-bacteria interactions to contrast their protective and pathogenic functions.

Bevins and colleagues demonstrated that HD5-transgenic mice are markedly resistant to oral challenge with virulent *Salmonella typhimurium*, consistent with the antibacterial activity of HD5 *in vitro*, whereas wild-type mice are susceptible to infection (81). An *in vivo* protective role against *Salmonella* infection is also illustrated for mouse intestinal α-defensins or cryptdins (82). Of note, enteric HD6, while exhibiting little bactericidal and membranolytic activity *in vitro*, protects mice from *Salmonella* infection by entrapping bacteria with a unique self-assembled “nanonets” structure to preclude the pathogen’s direct contact with the intestinal epithelium (83).

HNP1–3 secreted by infiltrating neutrophils in *Staphylococcus aureus* infection induce TNF-α and IFN-γ release from macrophages, which, in turn, increase phagocytosis of pathogens by macrophages – an essential step in bacterial clearance (84, 85). HNP1 also inhibits phagosomal escape and intracellular multiplication of *Listeria monocytogenes* and *Mycobacterium tuberculosis* in macrophages (86, 87), suggesting that the defensin, although not being expressed by macrophages, contributes to their antimicrobial function. Notably, HNP1 acts in the aftermath of *Salmonella* infection as a “molecular brake” on macrophage-driven inflammation by preventing protein translation to ensure both pathogen clearance and the resolution of inflammation with minimal bystander tissue damage (88).

**FIGURE 2:**
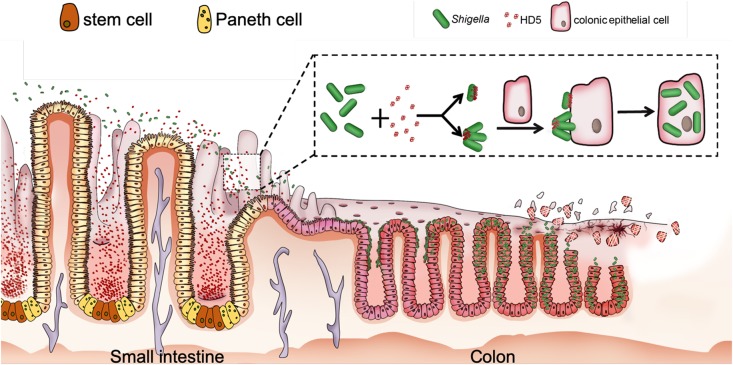
Proposed model for HD5-promoted *Shigella* infection of the colonic epithelium. HD5 in the lumen of the small intestine encounters poorly adhesive *Shigella* in transit, binds to *Shigella* surface, and promotes *Shigella* adhesion to the colonic epithelium by bridging single bacterium and host cell and/or clustering multiple bacteria for multivalent attachment to host cell, leading to increased bacterial infection from the apical surface (89, 91).

While the protective roles of defensins in bacterial infection are widely reported in the field, we have made a surprising recent discovery that α-defensins can contribute to the pathogenicity of *Shigella* (89–92). Unlike other enteropathogenic bacteria, *Shigella* lacks general adhesion machinery such as fimbriae due presumably to pervasive genome reduction during the course of adaptation to the intracellular environment (93–95). As a result, *Shigella* is much less adhesive and invasive *in vitro* than other fimbriated enteropathogenic bacteria despite its extraordinary infectivity in humans. Further, although highly infectious in humans, *Shigella* hardly infects any other animals including mice with abundant enteric α-defensins (cryptdins) (96, 97). This seemingly paradoxical phenomenon or conundrum in *Shigella* pathogenesis has remained largely obscure mechanistically at the molecular and cellular levels (97–99). We found that the lack of fimbriae in *Shigella* affords the pathogen a unique bacterial surface, onto which HD5 forms multimeric structures to mediate *Shigella* adhesion to host epithelium; enhanced bacterial adhesion in turn strongly promotes *Shigella* invasion of host cells, ensuing dramatically augmented infection *in vivo* and *ex vivo* (Figure 2). These studies support the premise that *Shigella* exploits HD5 for virulence (89, 91), thereby explaining not only its extraordinary pathogenicity but also its restricted host selectivity.

HNP1 is also active in promoting *Shigella* infection of epithelial cells (90), consistent with an earlier finding that human neutrophil granular proteins (containing HNPs) enhance *Shigella* adhesion *in vitro* at sub-lethal concentrations (100). Although HNP1 is weaker than HD5 with respect to their ability to promote *Shigella* adhesion, its strong activity in disrupting the epithelial barrier contributes additionally to *Shigella* infection (90). It is worth noting that HD5 exacerbates the pathogenicity of *Shigella* in macrophages. Despite that HD5 boosts phagocytosis of *Shigella* by macrophages, an antimicrobial event generally unfavorable to invading pathogens, it fails to prevent subsequent phagosomal escape and intracellular multiplication of *Shigella*, resulting in necrosis of infected macrophages induced by multiplying *Shigella* and massive release of intracellular bacteria (92).

For human α-defensins, their hydrophobicity and selective cationicity segregated on a dimeric structure stabilized by intramolecular disulfides are critical for antimicrobial activity (11). Several mutational studies have identified the functional determinants of α-defensins in promoting viral and bacterial infections (25, 56, 57, 59, 60, 90–92). Briefly, disulfide bonding in defensins is absolutely required for their ability to enhance HIV-1 infection (25, 60) and to promote *Shigella* adhesion and invasion (91, 92); hydrophobic residues in α-defensins, i.e., Trp26 and Phe28 in HNP1, Leu16, Leu26, Tyr27 and Leu29 in HD5, and Phe2 and Phe29 in HD6, play a pivotal functional role (59, 90, 91); dimerization and/or oligomerization of α-defensins are functionally indispensable (59, 83, 91, 101, 102); selective cationicity, as exemplified by Arg28 in HD5, can be critical for promoting HIV and *Shigella* infection (59, 91, 92). Obviously, although α-defensins are highly variable in amino acid sequence, their functional determinants are rather conserved, irrespective of their pathogenic and protective roles in host immunity.

## Defensins in Tumorigenesis

Most cancers develop from epithelial cells and tissues (carcinomas) where β-defensins are expressed for mucosal surface protection against microbial infection (26, 27, 103, 104). Since β-defensins are differentially expressed in normal tissues and tumors, their role in tumor development and progression has attracted considerable interest (32, 105–107). HBD1 is downregulated in most carcinomas (108–118), and the stimuli of this downregulation are yet to be identified. Growing evidence suggests that HBD1 functions as a tumor suppressor in most carcinomas (110, 119, 120). By contrast, HBD3 is frequently overexpressed in various carcinomas (121–124), and its upregulation has been ascribed to LPS-stimulated EGFR activation (121) or HPV co-infection-induced p53 degradation (125), among others. Importantly, HBD3 stimulates tumor growth and migration (122, 123, 126), confers resistance of tumor cells to apoptosis (127), and helps the recruitment of tumor-associated macrophages that promote tumor progression (127, 128). Consistent with the oncogenic role of upregulated H BD3, mouse β-defensin 14, the ortholog of HBD3, acts as a chemoattractant to enhance angiogenesis and tumor development *in vivo* (129). The regulation of HBD2 and its influence in tumorigenesis vary from cancer to cancer (106) and can be controversial at times (130, 131). HBD2 is upregulated in esophageal, lung and skin cancers (108, 109, 118, 132), but downregulated in oral and colon cancers (112, 114, 133). While the mechanisms of HBD2 regulation are only partially understood (132, 134, 135), HBD2 appears to play a suppressive role in tumor development and progression when it is downregulated (136), but a proliferative role when upregulated (131, 132, 137–139), in agreement with HBD1 and HBD3. The suppressive and proliferative properties of defensins in tumorigenesis are tabulated in Table 1.

**TABLE 1:** Suppressive and proliferative properties of human defensins in tumorigenesis.

Beneficial (tumor-suppressing)				Detrimental (tumor-promoting)			
Defensins	Cancers	Mechanisms	Refs	Defensins	Cancers	Mechanisms	Refs
HBD1	Bladder	Inhibiting growth	(110, 119)	HBD3	Oral	Stimulating growth	(122, 123, 126–
	Renal	promoting apoptosis	(110)		neck and head	promoting migration	128)
	prostate	inhibiting migration	(120)		cervical	trafficking TAM	(127; 128)
	oral					resisting apoptosis	(127)
*HBD2*	*Oral*	*Inhibiting growth and invasion*	(136)	HBD2	Esophageal	Stimulating growth	(131, 132, 139)
					Lung	promoting angiogenesis	(137)
					Cervical		
HNP1–3	Colorectal	Direct cytolysis (high concentration)	(157)	HNP1–3	Renal	Stimulating growth (low concentration)	(150)
	lung	inducing apoptosis	(158–160)		bladder	promoting invasiveness	(152; 153)
	bladder	inhibiting angiogenesis	(160–162)		oral		
	renal	reversing immune alteration	(163)				
	neck and head						
	oral						

The role of α-defensins in tumorigenesis has also been extensively examined (140, 141). Elevated levels of myeloid α-defensins, HNP1–3, are frequently detected in many different types of tumor tissues and in biological fluids from cancer patients (142–155). While tumor-infiltrating immune cells, and neutrophils in particular, are likely a major contributor to increased HNP1–3 in tumors (151), several studies also suggest that tumor cells themselves may produce HNP1–3 through a yet-to-be-identified mechanism (142, 150). HNP1–3 have been shown to promote tumor cell proliferation (150, 156), contributing to tumor progression and invasiveness (152, 153). Due to their membranolytic activity toward bacteria and limited sites of expression, much of the early studies of α-defensins have focused on their ability to lyse tumor cells at high concentrations (157). More recent work, however, has shed light on the mechanistic complexity of the antitumor activity of HNP1–3, including inducing apoptosis (158–160), inhibiting angiogenesis (160–162), and altering immune milieu in HPV-associated neoplasia by recruiting immature dendritic cells (163).

## Concluding Remarks

Long recognized as a class of host defense peptides and immunomodulators important for innate immune responses to viral, bacterial and fungal infections, human defensins are widely thought to be host protective. Growing recent evidence suggests, however, that they can also be pathogenic under certain biological conditions by promoting viral and bacterial infections. The interchangeable roles between a “Swiss army knife” and a “double-edged sword” played by human α-defensins in host immunity are under-appreciated in the field, despite the well-recognized fact that defensins can be both suppressors and promotors in tumorigenesis, depending on which defensin and cancer type are studied. While the mechanisms of host protection by human defensins are well-understood, much remain obscure with respect to the molecular and cellular events dictating defensins’ pro-infective activity. A better understanding of how human defensins promote infection may ultimately lead to new therapeutic interventions of infectious diseases.

## Author Contributions

DX and WL wrote the manuscript.

## Conflict of Interest

The authors declare that the research was conducted in the absence of any commercial or financial relationships that could be construed as a potential conflict of interest.
